# Supplementary material for: Hijacking the Mustard-Oil Bomb: How a Glucosinolate-Sequestering Flea Beetle Copes With Plant Myrosinases
Source: Front Plant Sci. 2021 May 20;12:645030. doi: 10.3389/fpls.2021.645030 (PMC8173161; doi:10.3389/fpls.2021.645030)
Supplement: Supplementary file 1 [file Data_Sheet_1.pdf]

Supplementary Material

1 Supplementary Methods and Results

1.1 Chemical analyses of *Arabidopsis* wild type and *tgg* plants

For chemical analyses of *Arabidopsis* wild type and *tgg* plants, we harvested whole rosettes of six-week old plants (n = 10). Rosettes were frozen in liquid nitrogen, freeze-dried and homogenized to plant powder by shaking with metal beads (2.4 mm diameter, Askubal) for 2 min at 25 Hz in a TissueLyser II (Qiagen, Hilden, Germany). Glucosinolates were extracted from 20 mg plant powder, converted to desulfo-glucosinolates and analyzed by HPLC-DAD as described in Beran et al. (2014). To determine the soluble protein content, 10 mg plant powder were extracted with 900 µL of 20 mM MES buffer pH 6.5 by shaking with metal beads for 2 min at 20 Hz in a TissueLyser II, followed by centrifugation at 4 °C for 10 min at 16,000 × g. Protein levels in supernatants were determined using the Bradford protein assay (Bio-Rad, Munich, Germany). Amino acids and sugars were analyzed by LC-MS/MS. Therefore, 15 mg plant powder were extracted with 1 mL of 80% MeOH by shaking and centrifugation as described above. The supernatant was diluted 1:10 with water containing a mix of $^{15}N/^{13}C$ labeled algal amino acids at a concentration of 10 µg × mL^{-1} (Isotec, Miamisburg, OH, USA). Amino acids were analyzed by LC-MS/MS on a Zorbax Eclipse C18-column (XDB-C18, 50 x 4.6 mm x 1.8 µm; Agilent, Santa Clara, CA, USA) (for details, refer to Crocoll et al. (2016)). Each amino acid was quantified relative to the peak area of its corresponding labeled amino acid, except for tryptophan (using phenylalanine and applying a response factor of 0.42) and asparagine (using aspartate and a response factor of 1.0). Soluble sugars were analyzed from the 1:10-diluted extract by LC-MS/MS on a hydrophilic interaction liquid chromatography (HILIC)-column (apHera-NH_2 Polymer; 15 × 4.6 mm, 5 µm; Supelco, Bellefonte, PA, USA) as described in Madsen et al. (2015). Sugars were quantified using external standard curves prepared from authentic standards of glucose, fructose, sucrose (Sigma-Aldrich, Steinheim, Germany) and raffinose (Fluka, Seelze, Germany).

1.2 Partial purification of myrosinase from *Sinapis alba*

We purified commercially available myrosinase enzyme (100 units (U), isolated from *Sinapis alba* seeds; Sigma-Aldrich) by fast protein liquid chromatography. The crude enzyme extract (ca. 25 U) was dissolved in 1 mL buffer (20 mM Tris·HCl, 0.15 M NaCl, pH 8, containing protease inhibitors (cOmplete, EDTA-free, Roche, Mannheim, Germany)) and subjected to size exclusion chromatography using a Superdex 200 10/300 GL column (GE Healthcare, Munich, Germany) as described in Beran et al. (2014). Fractions were tested for myrosinase activity as described below. Active fractions were pooled and desalted using Zeba Spin Desalting Columns (7 kDa MWCO 5 mL; Thermo Fisher Scientific, Bremen, Germany) equilibrated with 20 mM Tris HCl pH 8 before anion exchange chromatography using a 1 mL ResourceQ column (GE Healthcare) as described in Beran et al. (2014). Fractions containing myrosinase activity were pooled and dialyzed overnight against 20 mM MES buffer pH 6.5 containing 20% (v/v) glycerol at 4 °C using a Slide-A-Lyzer Dialysis Cassette (10 kDa MWCO, 3 mL, Thermo Fisher Scientific). After dialysis,

protease inhibitors (cOmplete, EDTA-free, Roche) were added, the protein concentration determined using the Bradford protein assay (Bio-Rad) and the extract was stored at 4 °C.

Protein fractions were screened for myrosinase activity using a protocol modified from Travers-Martin et al. (2008). Assays consisted of 5 µL sample, 45 µL 20 mM MES buffer (pH 6.5) containing 2.78 mM allyl glucosinolate (Carl Roth, Mannheim, Germany) as substrate, and 50 µL assay reagent (20 mM MES buffer (pH 6.5) containing 57 U/ml glucose-oxidase (E.C.1.1.3.4, from *Aspergillus niger*, Serva, Heidelberg, Germany), 5.6 U/ml peroxidase (E.C.1.11.1.7, from horseradish, Serva), 30.7 mM phenol (Sigma-Aldrich) and 2.8 mM 4-aminoantipyrine (Sigma-Aldrich)). Assays were incubated at room temperature for 30 min in transparent polystyrene 96-well microplates (Nunc, Thermo Fisher Scientific). Myrosinase activity was visually detected (pink assay color).

1.3 pH measurements of *P. armoraciae* gut homogenates

We dissected the midgut of adult *P. armoraciae* beetles collected from *B. juncea* rearing cages ($n = 6$). Dissected midguts (containing plant material) were homogenized individually in 40 µL deionized water and the pH of the obtained midgut homogenate was measured using an InLab Micro electrode (Mettler-Toledo, Schwerzenbach, Switzerland). The pH of midgut homogenates of *P. armoraciae* was 4.7 ± 0.2 (mean ± SD).

1.4 Myrosinase inhibition assays with *Psylliodes chrysocephala* gut content

The cabbage stem flea beetle, *Psylliodes chrysocephala*, can sequester glucosinolates from its host plants but most ingested glucosinolates appear to be hydrolyzed during feeding and digestion (Beran et al., 2018). We tested whether gut content extracts reduce plant myrosinase activity in *in vitro* assays as described for *P. armoraciae* beetles. *P. chrysocephala* was reared on three- to four-week old potted *Brassica rapa* plants as described in Beran et al. (2018). The gut content of newly emerged adult beetles was isolated as described for *P. armoraciae* and myrosinase inhibition assays with untreated and boiled gut extracts were performed according to the description in the main text ($n = 4$).

Supplementation of *S. alba* myrosinase with untreated and boiled gut extracts significantly reduced plant myrosinase activity *in vitro* by up to 44% (Supplementary Figure 1, One-way ANOVA, $F = 75.965$, $p < 0.001$). In contrast to assays performed with *P. armoraciae* gut extracts (Figure 4B), the effect of untreated and boiled extracts did not differ in *in vitro* assays with *P. chrysocephala* gut extracts.

1.5 Performance experiment with *P. armoraciae* larvae

We performed a long-term feeding experiment to investigate the influence of plant myrosinase activity on developmental time, fresh weight, and the energy budget of *P. armoraciae* larvae. Early second instar larvae were randomly assigned to either wild type or *tgg* plants, and were regularly provided with fresh leaves until they had developed into prepupae. We recorded the developmental time and the final weight of each individual (n = 68-74), froze the prepupae in liquid nitrogen, and stored them at -20 °C until analysis of energy reserves (performed as described in the main text; $n = 11$ per *Arabidopsis* genotype, two individuals per replicate). The food plant had no influence on

developmental time, fresh weight, and energy reserves of *P. armoraciae* larvae (Supplementary Table 7).

1.6 Sequestration experiment with *P. armoraciae* larvae

Previous experiments suggested that *P. armoraciae* larvae are not able to prevent hydrolysis of ingested glucosinolates from *Arabidopsis* wild type leaves (Sporer et al., 2020). To investigate the influence of plant myrosinase activity on the metabolic fate of ingested glucosinolates in *P. armoraciae* larvae, we performed a feeding experiment with *Arabidopsis* wild type, *tgg* and *myb* plants, and quantified the levels of 4MSOB glucosinolate and its hydrolysis products in larvae and feces. The experimental set-up followed *Experiment 2* described in the main text, except that the midribs of *Arabidopsis* leaves were removed using a scalpel to prevent larvae from mining. Feces, larvae and remaining leaves were sampled, extracted and analyzed by LC-MS/MS (for 4MSOB glucosinolate and derived metabolites) as described in *Experiment 2* ($n = 5$, 5 larvae per replicate). The levels of 4MSOB glucosinolate in fed leaves did not differ significantly (Student's t-test, $t = 0.132$, $p = 0.898$). The detected amounts of 4MSOB glucosinolate and its hydrolysis products in larvae and feces are summarized in Supplementary Table 8.

In agreement with previous data, *tgg*-fed larvae sequestered higher levels of 4MSOB glucosinolate than wild type-fed larvae (Supplementary Table 8, Sporer et al. (2020)). Because the total levels of glucosinolate-derived metabolites differed greatly between replicates, we compared their relative composition between treatments (Supplementary Table 8, results of statistical analyses are summarized in Supplementary Table 1). This comparison revealed a food plant-dependent composition of glucosinolate-derived metabolites in larval bodies. In wild type-fed larvae we detected predominantly 4MSOB cyanide, whereas 4MSOB glucosinolate, 4MSOB isothiocyanate and 4MSOB isothiocyanate-derived metabolites were more abundant in *tgg*-fed larvae (Supplementary Figure 2). Feces of wild type- and *tgg*-fed larvae contained similar percentages of mainly 4MSOB glucosinolate and 4MSOB cyanide (Supplementary Table 1). Overall, glucosinolate hydrolysis accounted for 95% and 76% of the total detected metabolites in wild type- and *tgg*-fed larvae, respectively (bodies and feces). These results indicate that *P. armoraciae* larvae have a much higher glucosinolate turnover rate compared to adults (Figure 2B), and that this turnover is largely independent of plant myrosinase activity.

2 Supplementary Figures and Tables

2.1 Supplementary Tables

Supplementary Table 1. Methods and results of statistical analyses.

Experiment	Comparison	Statistical method	Variable	Statistics	*p*
Experiment 1	Relative 4MSOB and 4MTB glucosinolate accumulation in beetles (wild type vs. *tgg*)	Mann-Whitney rank sum test	Percentage	*U* = 482.000	< 0.001
	Beetle feeding rate (cm^2) (wild type vs. *tgg*)	Student's *t*-test	Leaf area	*t* = 0.592	0.564
	Plant 4MSOB and 4MTB glucosinolate levels (wild type vs. *tgg*)	Mann-Whitney rank sum test	Concentration	*U* = 390.000	0.980
	Total glucosinolate levels in leaves (wild type vs. *tgg*)	Mann-Whitney rank sum test	Concentration	*U* = 794.000	0.954
Experiment 2	Percentage of 4MSOB glucosinolate hydrolysis products relative to the total detected amount of 4MSOB glucosinolate and its hydrolysis products in beetles (wild type vs. *tgg*)	Student's *t*-test	Percentage	*t* = 2.754	0.025
	Percentage of 4MSOB glucosinolate hydrolysis products relative to the total detected amount of 4MSOB glucosinolate and its hydrolysis products in feces (wild type vs. *tgg*)			*t* = 7.229	< 0.001
	4MSOB glucosinolate and derived metabolites in beetles (wild type vs. *tgg*)	Student's *t*-test, Mann-Whitney rank sum test	Amount	see Supplementary Table 2	
	4MSOB glucosinolate and derived metabolites in beetle feces (wild type vs. *tgg*)				
	4MSOB glucosinolate levels in leaves (wild type vs. *tgg*)	Student's *t*-test	Concentration	*t* = 0.371	0.720
Experiment 3	Recovery of ingested allyl glucosinolate in beetles and feces (wild type vs. *tgg*)	Student's *t*-test	Percentage[A]	see Table 1	
Experiment 4	Emitted allyl isothiocyanate per beetle (allyl glucosinolate-spiked wild type vs. *tgg*)	Mann-Whitney rank sum test	Amount	*U* = 0.000	0.001
	Sequestered allyl glucosinolate per beetle (allyl glucosinolate-spiked wild type vs. *tgg*)	Student's *t*-test	Amount	*t* = 1.689	0.119
Long term feeding experiment	Weight, soluble protein and other metabolites (wild type vs. *tgg*)	Student's *t*-test, Mann-Whitney rank sum test	Concentration	see Supplementary Table 5	
Glucosinolate distribution shortly	4MSOB glucosinolate level in beetle gut (wild type vs. *tgg*)	Student's *t*-test	Percentage[A]	*t* = 0.161	0.880
	Plant myrosinase inhibition by gut extracts	One-way ANOVA	Activity	*F* = 85.639	< 0.001

Experiment	Comparison	Statistical method	Variable	Statistics	*p*
after feeding and inhibition assays					
Myrosinase activity in feces	Ingested and excreted myrosinase activity	Paired *t*-test[B]	Activity	$t = 10.449$	< 0.005
	Co-incubation of plant myrosinases with feces homogenates	Paired *t*-test		$t = 0.158$	1.000
Supplementary myrosinase inhibition assays with *Psylliodes chrysocephala* gut content extracts	Plant myrosinase inhibition by gut extracts	One-way ANOVA	Activity	$F = 75.965$	< 0.001
Supplementary feeding experiment with larvae	Percentage of 4MSOB glucosinolate hydrolysis products relative to the total detected amount of 4MSOB glucosinolate and its hydrolysis products in larvae (wild type vs. *tgg*)	Mann-Whitney rank sum test	Percentage	U = 0.000	0.008
	Percentage of 4MSOB glucosinolate hydrolysis products relative to the total detected amount of 4MSOB glucosinolate and its hydrolysis products in feces (wild type vs. *tgg*)	Student's *t*-test		$t = 2.907$	0.020
	4MSOB glucosinolate and derived metabolites in larvae (wild type vs. *tgg*)	Student's *t*-test, Mann-Whitney rank sum test	Amount	see Supplementary Table 8	
	4MSOB glucosinolate and derived metabolites in larval feces (wild type vs. *tgg*)			see Supplementary Table 8	
	Plant 4MSOB glucosinolate levels (wild type vs. *tgg*)	Student's *t*-test	Concentration	$t = 0.132$	0.898
Supplementary long term feeding experiment with larvae	Larval development time (wild type vs. *tgg*)	Mann-Whitney rank sum test	Days	$U = 2{,}319.500$	0.420
	Prepupal weight and energy reserves (wild type vs. *tgg*)	Student's *t*-test, Mann-Whitney rank sum test	Fresh weight or concentration	see Supplementary Table 9	

Wild type, *Arabidopsis* wild type; *tgg*, *Arabidopsis tgg;* [A]Arcsin-square-root transformed; [B]*p*-values adjusted for false discovery rate in multiple hypothesis testing with Benjamini–Hochberg method (Benjamini and Hochberg, 1995).

Supplementary Table 2. Amounts of 4MSOB glucosinolate and 4MSOB glucosinolate-derived hydrolysis products in adult *P. armoraciae* beetles and feces after one day feeding on *Arabidopsis* wild type and *tgg* leaves

	nmol per individual (mean ± SD; *n* = 5)							
	Beetle				Feces			
Metabolite	wild type-fed	*tgg*-fed	Statistics	*p*	wild type-fed	*tgg*-fed	Statistics[2]	*p*
4MSOB glucosinolate	3.734 ± 2.862	6.229 ± 2.953	*t* = 1.213	0.260	0.129 ± 0.166	0.867 ± 0.917	*U* = 5.000	0.151
4MSOB cyanide	0.483 ± 0.208	0.050 ± 0.048	*U* = 1.000	0.016	0.809 ± 0.460	0.078 ± 0.054	*U* = 1.000	0.016
4MSOB isothiocyanate	0.025 ± 0.020	0.006 ± 0.002	*U* = 4.000	0.095	0.040 ± 0.028	0.002 ± 0.001	*U* = 0.000	0.008
Other 4MSOB isothiocyanate-derived metabolites[1]	0.048 ± 0.025	0.008 ±0.004	*U* = 2.000	0.032	0.073 ± 0.038	0.013 ±0.017	*t* = 2.882	0.020
Total	4.290 ± 3.058	6.292 ± 2.980	*t* = 0.938	0.376	1.051 ± 0.639	0.960 ± 0.900	*t* = 0.166	0.873

[1]comprise 4MSOB isothiocyanate-glutathione conjugate, 4MSOB isothiocyanate-cysteinylglycine conjugate, 4MSOB isothiocyanate-cysteine conjugate. 2-(4-(methylsulfinyl)butylamino)-4,5-dihydrothiazole-carboxylic acid, 4MSOB amine, 4MSOB acetamide; [2]data were compared using Student's *t*-test or Mann-Whitney rank sum test.

Supplementary Table 3. Glucosinolate profile of newly emerged *P. armoraciae* adults reared on *B. rapa*.

Glucosinolate	nmol × mg FW^{-1} (mean ± SD; *n* = 20)
3-Butenyl	0.48 ± 0.53
4-Pentenyl	0.90 ± 0.68
2-Hydroxy-3-butenyl	3.66 ± 1.65
2-Hydroxy-4-pentenyl	0.82 ± 0.54
5-Methythiopentyl	1.53 ± 0.54
Benzyl	0.19 ± 0.14
2-Phenylethyl	0.35 ± 0.30
Indol-3-ylmethyl	0.23 ± 0.11
4-Methoxyindol-3-ylmethyl	0.06 ± 0.03
1-Methoxyindol-3-ylmethyl	0.22 ± 0.08
Total	8.42 ± 3.26

Supplementary Table 4. Levels of nutrients and glucosinolates in rosette leaves of six-week old *Arabidopsis* wild type and *tgg* plants (n = 10; mean ± SD).

		wild type	*tgg*	Statistics	*p*
Soluble protein (mg × g dry weight^{-1})		37.010 ±6.028	36.805 ± 7.269	t = 0.069	0.946
Amino acids (µmol × g dry weight^{-1})	Alanine	8.040 ± 1.418	8.016 ± 1.365	t = 0.038	0.970
	Arginine*	1.498 ± 0.509	1.207 ± 0.187	U = 30.000	0.140
	Asparagine	9.540 ± 2.831	8.687 ± 1.458	U = 44.000	0.678
	Aspartic acid	6.433 ± 0.764	6.862 ± 0.627	t = 1.302	0.209
	Glutamic acid	49.035 ± 5.004	50.804 ± 4.426	t = 0.794	0.437
	Glutamine	100.721 ± 17.043	109.767 ± 13.346	t = 1.254	0.226
	Histidine*	2.741 ± 0.341	2.909 ± 0.392	t = 0.968	0.346
	Isoleucine*	1.100 ± 0.188	1.041 ± 0.093	t = 0.847	0.408
	Leucine*	0.959 ± 0.128	0.898 ± 0.134	t = 0.991	0.335
	Lysine*	0.781 ± 0.088	0.747 ± 0.082	t = 0.836	0.414
	Methionine*	0.253 ± 0.022	0.237 ± 0.035	t = 1.185	0.252
	Phenylalanine*	0.800 ± 0.226	0.727 ± 0.092	U = 43.000	0.623
	Proline	3.984 ± 1.007	4.906 ± 1.017	U = 18.000	0.017
	Serine	16.839 ± 2.716	16.025 ± 0.996	U = 45.000	0.734
	Threonine*	11.012 ± 1.184	11.675 ± 0.850	t = 1.365	0.189
	Tryptophane*	0.139 ± 0.039	0.130 ± 0.016	U = 46.000	0.791
	Tyrosine	0.457 ± 0.087	0.392 ± 0.042	t = 2.013	0.059
	Valine*	1.820 ± 0.265	1.764 ± 0.128	t = 0.562	0.581
	Total essential amino acids*	21.103 ± 2.171	21.336 ± 1.176	t = 0.283	0.781
	Total non-essential amino acids	185.508 ± 23.637	196.770 ± 15.005	t = 1.207	0.243
	Total amino acids	206.611 ± 24.816	218.107 ± 15.327	t = 1.182	0.252
Soluble sugars (mg × g dry weight^{-1})	Glucose	0.154 ± 0.045	0.152 ± 0.044	t = 0.090	0.929
	Fructose	0.069 ± 0.019	0.049 ± 0.011	t = 2.796	0.012
	Sucrose	0.125 ±0.027	0.145 ± 0.016	t = 2.186	0.042
	Raffinose	0.080 ± 0.021	0.087 ± 0.009	U = 45.000	0.307
	Total sugars	0.428 ± 0.079	0.433 ± 0.060	t = 0.538	0.597
Glucosinolates (µmol × g dry weight^{-1})	3MSOP glucosinolate	1.924 ± 0.251	1.834 ± 0.130	U = 38.000	0.385
	4MSOB glucosinolate	15.071 ± 1.537	14.811 ± 1.253	t = 0.393	0.699
	5MSOP glucosinolate	0.510 ± 0.047	0.513 ± 0.042	t = 0.146	0.885
	7MSOH glucosinolate	0.249 ± 0.032	0.231 ± 0.016	t = 1.543	0.140
	8MSOO glucosinolate	1.209 ± 0.094	1.263 ± 0.082	t = 1.298	0.211
	4MTB glucosinolate	1.200 ± 0.132	1.183 ± 0.126	t = 0.285	0.779
	4OHI3M glucosinolate	0.022 ± 0.011	0.016 ± 0.010	t = 1.216	0.240
	I3M glucosinolate	1.756 ± 0.218	1.532 ± 0.138	t = 2.598	0.018
	4MOI3M glucosinolate	0.394 ± 0.035	0.368 ± 0.039	U = 28.000	0.104
	1MOI3M glucosinolate	0.088 ± 0.035	0.127 ± 0.105	U = 37.000	0.345
	Total glucosinolates	22.422 ± 1.902	21.877 ± 1.414	t = 0.689	0.500

*, essential amino acids for insect nutrition (Behmer, 2005); 3MSOP, 3-methylsulfinylpropyl; 4MSOB, 4-methylsulfinylbutyl; 5MSOP, 5-methylsulfinylpentyl; 7MSOH, 7-methylsulfinylheptyl; 8MSOO, 8-methylsulfinyloctyl; 4MTB, 4-methylthiobutyl; 4OHI3M, 4-hydroxyindol-3-ylmethyl; I3M, indol-3-ylmethyl; 4MOI3M, 4-methoxyindol-3-ylmethyl; 1MOI3M, 1-methoxyindol-3-ylmethyl; statistical analysis was performed using Student's *t*-test or Mann-Whitney rank sum test.

Supplementary Table 5. Fresh weight and energy reserves of newly emerged *P. armoraciae* adults after feeding on *Arabidopsis* wild type and *tgg* leaves for 10 days (mean ± SD).

Parameter	Sex	*n*	wild type-fed	*tgg*-fed	Statistics	*p*
mg Fresh weight	male	8-9	2.12 ± 0.19	2.10 ± 0.23	*t* = 0.212	0.835
	female	10	2.94 ± 0.21	2.80 ± 0.42	*t* = 0.891	0.385
µg Soluble protein × mg FW^{-1}	male	8-9	52.20 ± 6.65	51.36 ± 3.87	*U* = 34.000	0.885
	female	10	69.59 ± 15.03	77.15 ± 11.19	*t* = 1.210	0.242
µg Total lipids × mg FW^{-1}	male	8-9	1.34 ± 1.55	1.86 ± 3.16	*U* = 34.000	0.885
	female	10	4.61 ± 2.57	5.45 ± 3.29	*t* = 0.604	0.554
µg Glycogen × mg FW^{-1}	male	8-9	22.07 ± 5.59	26.72 ± 10.22	*U* = 27.000	0.413
	female	10	17.22 ± 5.38	16.70 ± 3.46	*t* = 1.099	0.286
µg Soluble carbo-hydrates × mg FW^{-1}	male	8-9	3.79 ± 2.87	2.77 ± 2.12	*U* = 25.000	0.312
	female	10	3.75 ± 1.08	3.06 ± 1.53	*U* = 47.000	0.850

Statistical analysis was performed using Student's *t*-test or Mann-Whitney rank sum test.

Supplementary Table 6. *Arabidopsis* TGG1-derived peptides detected by nano-UPLC-MS^E in feces of *P. armoraciae* adults fed with *Arabidopsis* wild type leaves.

Amino acid sequence	sample number[1]	Sequence identity
LFNSGNFEK	20	TGG1
GFIFGVASSAYQVEGGR	20	TGG1
GLNVWDSFTHR	20	TGG1
GGADLGNGDTTCDSYTLWQK	19	TGG1
FSIAWSR	19, 20	TGG1 and TGG6
YYNGLIDGLVAK	19, 20	TGG1
NWITINQLYTVPTR	19, 20	TGG1
GYALGTDAPGR	19, 20	TGG1 and TGG2
DDQKGMIGPVMITR*	19	TGG1
GMIGPVMITR	19, 20	TGG1
WFLPFDHSQESK	19, 20	TGG1
LPEFSETEAALVK	19, 20	TGG1
GIYYVMDYFK	19, 20	TGG1
TTYGDPLIYVTENGFSTPGDEDFEK	20	TGG1

[1]corresponding to sample number in Figure 4A; *contains one missed tryptic cleavage site.

Supplementary Table 7. Fresh weight and energy reserves of *P. armoraciae* prepupae reared on *A. thaliana* wild type and *tgg* leaves from the early second instar. Mean ± SD are listed.

Parameter	*n*	wild type-fed	*tgg*-fed	Statistics	*p*
Developmental time (days)	68-74	33 ± 2	34 ± 3	*U* = 2,319.500	0.420
mg FW × prepupa^{-1}	68-74	1.34 ± 0.23	1.32 ± 0.22	*t* = 0.331	0.741
µg Soluble protein × mg FW^{-1}	11	72.78 ± 6.91	77.47 ± 13.06	*U* = 49.000	0.470
µg Total lipids × mg FW^{-1}	11	51.87 ± 3.42	41.77 ± 3.46	*t* = -1.586	0.129
µg Glycogen × mg FW^{-1}	11	157.58 ± 34.98	128.68 ± 17.56	*U* = 52.000	0.599
µg Soluble carbohydrates × mg FW^{-1}	11	55.52 ± 2.88	51.24 ± 4.96	*t* = 0.526	0.605

Statistical analysis was performed using Student's *t*-test or Mann-Whitney rank sum test.

Supplementary Table 8. Detected amounts of 4MSOB glucosinolate and derived metabolites in *P. armoraciae* larvae and feces after one day feeding on *Arabidopsis* wild type and *tgg* leaves.

	nmol per individual (mean ± SD; $n = 5$)							
	Larva				Feces			
Metabolite	wild type-fed	*tgg*-fed	Statistics	*p*	wild type-fed	*tgg*-fed	Statistics	*p*
4MSOB glucosinolate	0.001 ± 0.001	0.372 ± 0.290	$t = 2.558$	0.034	0.100 ± 0.078	0.215 ± 0.165	$t = 1.262$	0.242
4MSOB cyanide	0.684 ± 0.415	0.207 ± 0.130	$U = 5.000$	0.151	1.158 ± 0.628	0.537 ± 0.342	$t = 1.737$	0.121
4MSOB isothiocyanate	0.016 ± 0.008	0.728 ± 0.286	$U = 0.000$	0.008	0.013 ± 0.007	0.004 ± 0.003	$t = 2.231$	0.056
Other 4MSOB isothiocyanate-derived metabolites[1]	0.023 ± 0.011	0.334 ± 0.162	$U = 0.000$	0.008	0.051 ± 0.027	0.027 ± 0.030	$t = 1.193$	0.267
Total	0.724 ± 0.430	1.641 ± 0.763	$t = 2.093$	0.070	1.322 ± 0.709	0.784 ± 0.506	$t = 1.236$	0.251

[1]comprise 4MSOB isothiocyanate-glutathione conjugate, 4MSOB isothiocyanate-cysteinylglycine conjugate, 4MSOB isothiocyanate-cysteine conjugate, 2-(4-(methylsulfinyl)butylamino)-4,5-dihydrothiazole-carboxylic acid, 4MSOB amine, 4MSOB acetamide; statistical analysis was performed using Student's *t*-test or Mann-Whitney rank sum test.

2.2 Supplementary Figures

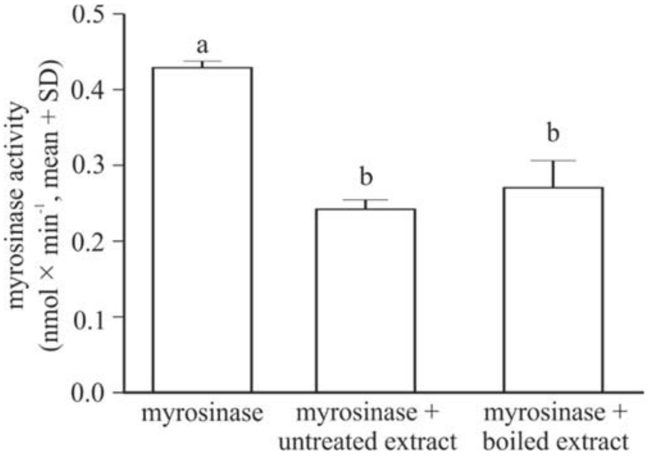

Supplementary Figure 1. *Psylliodes chrysocephala* gut content extracts reduce plant myrosinase activity *in vitro*. *In vitro* assays were performed by incubating partially purified *Sinapis alba* myrosinase with 4MSOB glucosinolate substrate and untreated or boiled gut content extracts from *P. chrysocephala* beetles (n = 4). Myrosinase activity was determined by quantifying the 4MSOB glucosinolate substrate in each assay after conversion to desulfo-glucosinolate and analysis by HPLC-DAD. Assays without myrosinase served as background controls and activities were subtracted from the corresponding samples. Different letters indicate significant differences, One-way ANOVA, $p < 0.01$. Statistical result is shown in Supplementary Table 1.

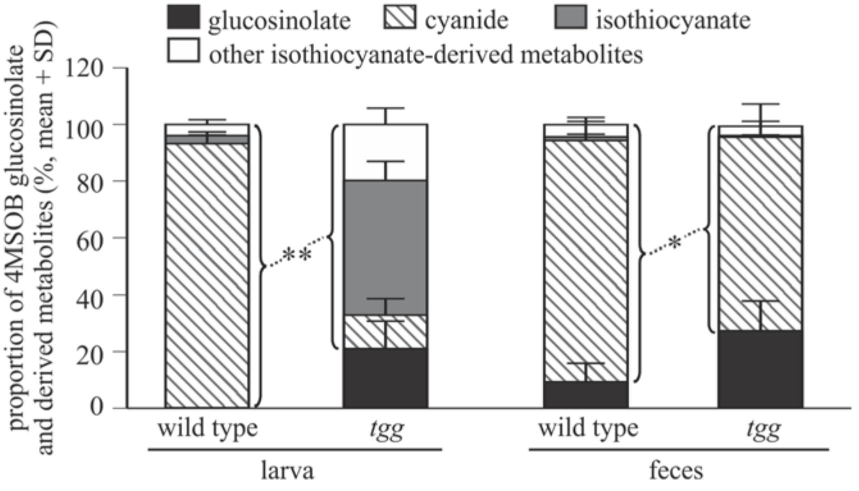

Supplementary Figure 2. Proportions of 4-methylsulfinylbutyl (4MSOB) glucosinolate and derived hydrolysis products detected in the body and feces of *P. armoraciae* larvae fed with *Arabidopsis* wild type or myrosinase-deficient *tgg* leaves. The relative composition of 4MSOB glucosinolate and hydrolysis products in bodies and feces of wild type- or *tgg*-fed larvae (n = 5) was determined after background subtraction from *Arabidopsis myb* fed control beetles (n = 5). Glucosinolates and hydrolysis products were extracted with 50% methanol and analyzed by LC-MS/MS. Detected amounts of metabolites were expressed relative to the total amounts of all detected metabolites derived from 4MSOB glucosinolate in larvae or feces (set to 100%). Dashed lines indicate significant differences between samples (larvae: Mann-Whitney rank sum test, $U = 0.000$, $p = 0.008$, feces: Student's t-test, $t = 2.907$, $p = 0.020$). Details of the statistical tests and results are shown in Supplementary Table 1. *, $p < 0.05$; **, $p < 0.01$; 4MSOB cyanide corresponds to the nitrile formed from 4MSOB glucosinolate. Other isothiocyanate-derived metabolites comprise 4MSOB isothiocyanate-glutathione conjugate, 4MSOB isothiocyanate-cysteinylglycine conjugate, 4MSOB isothiocyanate-cysteine conjugate, 2-(4-(methylsulfinyl)butylamino)-4,5dihydrothiazole-carboxylic acid, 4MSOB amine, and 4MSOB acetamide.

3 Supplementary References

Behmer, S.T. (2005). "Nutrition in Insects," in *Encyclopedia of Entomology*. (Dordrecht: Springer Netherlands), 1577-1582.

Benjamini, Y., and Hochberg, Y. (1995). Controlling the false discovery rate - a practical and powerful approach to multiple testing. *Journal of the Royal Statistical Society Series B-Statistical Methodology* 57**,** 289-300.

Beran, F., Pauchet, Y., Kunert, G., Reichelt, M., Wielsch, N., Vogel, H., Reinecke, A., Svatoš, A., Mewis, I., Schmid, D., Ramasamy, S., Ulrichs, C., Hansson, B.S., Gershenzon, J., and Heckel, D.G. (2014). *Phyllotreta striolata* flea beetles use host plant defense compounds to create their own glucosinolate-myrosinase system. *Proceedings of the National Academy of Sciences* 111**,** 7349-7354.

Beran, F., Sporer, T., Paetz, C., Ahn, S.-J., Betzin, F., Kunert, G., Shekhov, A., Vassão, D.G., Bartram, S., Lorenz, S., and Reichelt, M. (2018). One Pathway Is Not Enough: The Cabbage Stem Flea Beetle *Psylliodes chrysocephala* Uses Multiple Strategies to Overcome the Glucosinolate-Myrosinase Defense in Its Host Plants. *Frontiers in Plant Science* 9.

Crocoll, C., Mirza, N., Reichelt, M., Gershenzon, J., and Halkier, B.A. (2016). Optimization of engineered production of the glucoraphanin precursor dihomomethionine in *Nicotiana benthamiana*. *Front Bioeng Biotechnol* 4**,** 14.

Madsen, S.R., Kunert, G., Reichelt, M., Gershenzon, J., and Halkier, B.A. (2015). Feeding on leaves of the glucosinolate transporter mutant *gtr1gtr2* reduces fitness of *Myzus persicae*. *J Chem Ecol* 41**,** 975-984.

Sporer, T., Körnig, J., and Beran, F. (2020). Ontogenetic differences in the chemical defence of flea beetles influence their predation risk. *Functional Ecology* 34**,** 1370– 1379.

Travers-Martin, N., Kuhlmann, F., and Müller, C. (2008). Revised determination of free and complexed myrosinase activities in plant extracts. *Plant Physiol Biochem* 46**,** 506-516.
